# Notes and News

**Published:** 1888-11-10

**Authors:** 


### Notes and News.

Collected and Communicated.

Amongst other works of charity undertaken by the Whitworth executors, are two hospitals for Darley Dale, Derbyshire; one a general hospital, and the other for infectious cases. The cost will be about £20,000.

Many of our readers will share our regret at hearing that in consequence of the decision of the Court of Appeal on the Queen's Jubilee Hospital case, arrangements have been made to secure other premises close to those at Gloucester Terrace, which must be vacated. The multiplication of small special hospitals is to be condemned as inimicable to the best interests of the public and of the medical profession.

Underneath the bust of the late Dr. Fox, now erected in the Shire Hall at Taunton, is the following inscription: "Wilson Fox, M.D., F.R.S., Physician in Ordinary to her Most Gracious Majesty Queen Victoria. His talent commanded admiration, and his character won the esteem of all who knew him. Born at Wellington, December 2nd, 1831. Died May 3rd, 1887."

Hexham Local Board is divided as to the necessity of an infectious hospital. One member says that prevention is better than cure, and severely criticises the drainage scheme of the town. Another member thinks it is no use building a hospital till compulsory notification of infectious diseases is made the law. Some fine day, after the steed has been stolen the board will lock the stable door—that is, will build the hospital.

At the annual meeting of the Children's Hospital, Melbourne, Australia, held last month, it was stated that several of the cots, and one entire ward, were supported by the children who attended various public schools. The hospital has this year been enlarged, and a convalescent college secured in the country. This last ought to be a great boon for the hip disease cases which are so numerous in Melbourne, and who never recover so long as they remain in town.

A most amusing story comes from Manchester anent a late patient in the infirmary there. He was found apparently insensible in a train with no railway ticket in his pockets. He was removed to the infirmary where he recovered consciousness, but acted like a deaf mute. The doctors were sceptical and sent for a galvanic battery, but before they had time to apply it, the deaf mute sprang out of bed exclaiming, "My name is Smith, and you may put me down a rogue."

The Right Honourable the Lord Mayor will preside at the next sessional meeting of The Hospitals Association, to be held in the Great Hall of St. Bartholomew's Hospital, at half-past eight p.m., on Thursday, the 15th inst. Sir Sydney Waterlow, Bart., will read a paper on "Sixteen Years of the Hospital Sunday Fund: Its Influence on the Metropolitan Medical Charities." An interesting discussion on the questions raised by this paper is anticipated. Ladies have been specially included in the cards of invitation, and those who wish to be present to meet the new Lord Mayor should make immediate application to Mr. L. Broke Willoughby, Norfolk House, Norfolk Street, Strand, W.C.

There is a very pretty little cottage home at Roman Lodge, Brading, Isle of Wight, if any doctor or mother is looking out for a home in a mild climate for a sickly or afflicted child. So often when such a place is wanted it is not to be found, and just now at the beginning of winter many may be longing to know of such a home. The terms are moderate, and the matron (Mrs. Walker) is experienced, and has excellent references from doctors, the Duchess of Beaufort, and others.

On November 2nd the private chapel attached to St. Saviour's Hospital, Osnaburgh Street, was opened with an impressive service. The entire interior has been fitted up with the old oak which formed the choir of the Carthusian Convent of Busheim, which was dismantled a few years ago. The whole is the gift of Mr. Edward Howley Palmer. There is a service this (Saturday) afternoon, at five o'clock, when the public will be admitted to the chapel, and to the wards if they wish it.

The hearing of the action in connexion with the appointment of ophthalmic surgeon to Cardiff Infirmary was commenced last week before Justice Kekewich. In his opening statement Mr. Barber stated that the plaintiffs were members of a voluntary association known as the Glamorganshire and Monmouthshire Infirmary, and they were suing, on behalf of themselves and all other members or governors of the association, to get his lordship's declaration that at the election of an honorary ophthalmic surgeon in April, 1887, certain voting papers were improperly rejected, and that in consequence a gentleman named J. Tatham Thompson was improperly elected to the post. His lordship gave judgment in the defendant's favour. He held that the chairman of the meeting in April, 1887, was perfectly right in rejecting the 140 proxies tendered on behalf of Mr. Ensor, as they were wrong in form. He decided that Mr. Thompson had been properly elected, and dismissed the action, with costs.

A correspondent writes: "The awful events in Whitechapel must cause all thinking men and women to wish to help in creating a better state of things. What can be done, how can we help, who will start the question of pulling down all the dens and rookeries which exist, not only in Whitechapel, but in some of our loveliest seaside towns? It is of no use clergy and lady visitors preaching morality when the people to whom they preach 'live like pigs in a stye,' when boys and girls, married and single, live and sleep in one small room. It is no use preaching modesty and cleanliness, when there is no water to wash with, no place in which to wash in private. The wonder—as remarked a medical man who does much work amongst these dens—is that more of our young women do not go on the streets. They have no inducement to remain at home, and fresh air and company are easily found outside. A man remarked to us the other day, take the Bible in one hand and pull down these dens with the other, and put all common lodging-houses under the eye of the police, then preaching and teaching will be less of a mockery. Surely our Christianity is but an empty sound when we do not put our shoulders to the wheel, and anyhow one and all try to put away from amongst us this fearful curse which, like drunkenness, fills our hospitals, ruins our men and women body and soul, and costs our country untold gold. Difficulties there are without end, but were we less half-hearted, less selfish, we should realise that these poor, wretched women are indeed our sisters; that all classes, and both sexes, of whatever station, have much to answer for; and it is but mock modesty to put the subject on one side as a thing not to be mentioned in 'society.' Surely if we truly believe what we profess, we shall take up this work in some form or other. Anyhow, we shall not turn away from the subject, and in point of fact say, 'Am I my sister's keeper?'"

THE following vacancies are announced this week, the amount of salary in each case being given after the name of the institution :—

**Resident Medical Officers.**

Grantham Friendly and Trades Societies Medical Institution. Medical Officer. – £150, residence, etc.
Newcastle-upon-Tyne Borough Lunatic Asylum. Superintendent. —£450, residence, et·.
Lancashire County Asylum, at Rainhill. Medical Superintendent.—£1,000, residence, etc.

**Non-resident Medical Officers.**

City of London Hospital for Diseases of the Chest. Assistant Physician
Dental Hospital of London, Leicester Square. Lecturer on Mechanical Dentistry
Kensington Dispensary. Honorary Surgeon
Tobercurry Union. Medical Officer to Coolaney Dispensary.—£120.
Victoria University, Yorkshire College, Leeds. Joint Lecturer on Forensic Medicine.

---

A BOY named Geary, who was bitten near Armagh by a rabid dog last August, died on Saturday of hydrophobia. He had been treated by Mr. M'Gavern, known as the "Irish Pasteur," who had discharged him as cured. Dr. Fergus having certified that the boy died of hydrophobia, the coroner did not deem it necessary to hold an inquest.

---

THE following appointments have been made at the Great Northern Central Hospital: H. W. Syers, M.D., M.R.C.P., elected to the vacant office of physician to out-patients, caused by the promotion to the in-patient department of E. Clifford Beale, M.B., M.R.C.P., and C. E. H. Cotes, M.B., B.S., F.R.C.S., to the office of surgeon to out-patients, caused by the promotion to the in-patient department of C. B. Lockwood, F.R.C.S.

---

AT the annual meeting of the Grimsby and District Hospital subscribers, on October 25th, it was announced that ten out of the twenty-four beds necessary for the new "Veal" wing had been promised. Each bed costs £12 12*s.* Three beds are given by friendly societies, one by the Licensed Victuallers' Association of Grimsby, and others by Victoria, Countess of Yarborough, the Earl of Yarborough, Councillor Curry, Mrs. Grant-Thorold, &c. The Earl of Yarborough presided at the meeting, and congratulated the hospital on the present state of its finances.

---

THE *Graphic* of November 3rd gives pictures of three new hospitals which have been built in India, under the auspices of Lady Dufferin's Fund: The Walter Hospital, built by H.H. the Maharajah of Udaipur, Rajputana; a hospital and dispensary built by H.H. the Maharajah of Dharbunga; and the Dufferin Hospital at Nagpur, built by the Central Province Branch of the Fund. The two first are handsome edifices, worthy of their princely builders, the Nagpur Hospital is more modest in appearance, but being only one storey in height is perhaps better suited for the conditions of hospital life in a hot climate.

---

IT seems that the Leicester Infirmary is advancing with the times. During the present year a handsome memorial chapel, which will seat 100, has been presented to the infirmary by the friends and patients of the late Dr. Birch. Also a commodious porch has been erected at the principal entrance by the friends and patients of the late Dr. Pearce. The Leicester Infirmary was opened in the year 1771, it was partly rebuilt and enlarged in the years 1861-2 at a cost of £17,000. In 1887 an extension of the west wing took place, which provided 26 new bed-rooms for the nurses, with dining-rooms and sitting-rooms, together with four isolation wards for special cases; this cost £5,000. The daily number of patients in the house during last year was 172½. The resident staff consists of house surgeon, assistant house surgeon, two dressers, a lady superintendent (Miss Rogers) and thirty-eight nurses. In addition to this there are an honorary surgeon-dentist, chaplain, secretary, and others.

THE London Homœopathic Hospital announces a series of six private subscription dances, under the patronage of La Marquise de Saliceto, the Countess of Chichester, the Countess of Morley, the Countess Sydney, and other ladies. For these dances a ball-room and suite of rooms have been engaged at the Westminster Town Hall, and the arrangements are to be quite exceptional. The Anglo-Hungarian band (scarlet uniform) has been secured for the series. Subscribers can only be nominated upon the signed introduction of a patroness or steward.

---

A FURTHER step has been taken towards providing a suitable building in which to carry on the work of the charity known as the Bradford Hospital for the Sick Children of the Poor, the competition between five firms of local architects having resulted in the selection by the committee of the design sent in by Messrs. W. H. H. and J. E. Marten, of Manningham Lane. At present the work of this charity is conducted in a large house situate in Spring Gardens, where there are about twenty-four beds, which are generally occupied. The new building will be in the Queen Anne style, and will consist of two large wards to hold fifty beds, an isolation ward, and the usual offices.

---

COCAINE has claimed another victim in the person of a young Swiss physician. He began with morphine, which he eventually exchanged for cocaine. His first experiments were attended by increased vigour of mind. This, however, was soon followed by depression. After increased doses of the drug, he experienced hallucinations, and was at length left utterly sleepless and without appetite. To escape the fatal influence of the drug he gave up his practice and went as surgeon in a ship. At length, by means of long sea-voyages he overcame his craving, and was restored to health; only to fall a victim to the poison once more on finding it accessible. Persistent indulgence in the drug resulted in one of the most horrible of deaths—exhaustion from tetanus (lock-jaw).

---

C. G. C. writes: "May I remark upon Annotation, p. 75, 'Women and their Victims'? It is intensely severe, but scarcely unfair. Having lived and worked through life among and for women, I find the average want of capacity to understand broad principles most discouraging. They will be lazily kind and generous to the most unworthy objects, losing sight of the fact that they thereby often gravely injure or thwart honest efforts to improve or correct them. The extreme disproportion between the numbers of men and women in England seems to me to bear on the whole subject, because many women pass through life devoid of tangible duties and responsibilities, while mentally, as physically, the unused mental fibres wither and die out. Moreover, without accepting all Mrs. Longshore Potts' teaching, I feel sure that if young girls were simply taught the broad facts of human life and the special management their own health requires, much hysterical and emotional derangement would be averted, and their consequences must be recognised by every physician. A really healthy sensible woman of any age is one of the rarest outcomes of our 'emancipation,' but I think young girls of the present day, however silly, deserve great sympathy, as making or finding work is not quite the natural development of women's lives, and even to do this requires energy and resources. Nursing alone—where the vocation exists—seems to me capable of satisfying sensible and educated women, while bringing them face to face with reality and common-sense, both rather deficient factors in lady-like life. I think too many kind-hearted fathers object to their daughters working without necessity: that was where Mr. Garrett was so wise in employing his daughters in his own counting-house, and thus fitting them under shelter for wider work. I hope you will not disapprove of views I hold very strongly."

